# Molecular Cloning and Expression Analysis of Eight *PgWRKY* Genes in *Panax ginseng* Responsive to Salt and Hormones

**DOI:** 10.3390/ijms17030319

**Published:** 2016-03-04

**Authors:** Hao Xiu, Mohammed Nuruzzaman, Xiangqian Guo, Hongzhe Cao, Jingjia Huang, Xianghui Chen, Kunlu Wu, Ru Zhang, Yuzhao Huang, Junli Luo, Zhiyong Luo

**Affiliations:** Molecular Biology Research Center, State Key Laboratory of Medical Genetics, School of Life Sciences, Central South University, Changsha 410078, China; haoxiu1990@163.com (H.X.); nzaman_global@yahoo.com (M.N.); 142511013@csu.edu.cn (X.G.); caohongzhe1986@hotmail.com (H.C.); huangjj1985@126.com (J.H.); chenxianghui@163.com (X.C.); wukunlu@163.com (K.W.); zhangru2002@hotmail.com (R.Z.); 15399906281@163.com (Y.H.); jlluo@scripps.edu (J.L.)

**Keywords:** *Panax ginseng*, transcription factors, abiotic stress

## Abstract

Despite the importance of *WRKY* genes in plant physiological processes, little is known about their roles in *Panax ginseng* C.A. Meyer. Forty-eight unigenes on this species were previously reported as WRKY transcripts using the next-generation sequencing (NGS) technology. Subsequently, one gene that encodes PgWRKY1 protein belonging to subgroup II-d was cloned and functionally characterized. In this study, eight *WRKY* genes from the NGS-based transcriptome sequencing dataset designated as *PgWRKY2-9* have been cloned and characterized. The genes encoding WRKY proteins were assigned to WRKY Group II (one subgroup II-c, four subgroup II-d, and three subgroup II-e) based on phylogenetic analysis. The cDNAs of the cloned *PgWRKYs* encode putative proteins ranging from 194 to 358 amino acid residues, each of which includes one WRKYGQK sequence motif and one C_2_H_2_-type zinc-finger motif. Quantitative real-time PCR (qRT-PCR) analysis demonstrated that the eight analyzed *PgWRKY* genes were expressed at different levels in various organs including leaves, roots, adventitious roots, stems, and seeds. Importantly, the transcription responses of these *PgWRKYs* to methyl jasmonate (MeJA) showed that *PgWRKY2*, *PgWRKY3*, *PgWRKY4*, *PgWRKY5*, *PgWRKY6*, and *PgWRKY7* were downregulated by MeJA treatment, while *PgWRKY8* and *PgWRKY9* were upregulated to varying degrees. Moreover, the *PgWRKY* genes increased or decreased by salicylic acid (SA), abscisic acid (ABA), and NaCl treatments. The results suggest that the *PgWRKYs* may be multiple stress–inducible genes responding to both salt and hormones.

## 1. Introduction

WRKY transcription factors (TFs) are confined in plant kingdom and are essential for gene expression [1]. However, WRKY-like proteins were also reported in non-plant species such as *Giardia* *lamblia*, a primitive eukaryote, *Chlamydomonas* *reinhardtii*, a unicellular green alga, and *Dictyostelium* *discoideum*, a smile mold closely associated with the lineage of animals and fungi [2,3]. The name of “WRKY” was derived from the highly conserved WRKY domain region, a 60-amino-acid stretch containing one conserved WRKYGQK sequence motif at the N-terminal portion followed by one of the two types of zinc-finger motifs, the Cys_2_His_2_ or Cys_2_HisCys, at the C-terminal region. Both motifs of the domain exhibit high binding affinity to the W-box in the promoter of the target gene to modulate the rate of transcription [4,5]. All the known WRKY proteins are structurally divided into three distinct groups (Groups I, II, and III) on the basis of the number of WRKY domains and the pattern of the zinc-finger motif. Group I WRKY proteins typically carry two WRKY domains and a C_2_H_2_-type zinc-finger motif, where the C-terminal WRKY domain is considered to be active in DNA binding. Group II WRKY proteins consist of a single WRKY domain and a same type of zinc-finger motif such as in group I. They are further divided into five subgroups, a–e (II-a, II-b, II-c, II-d, and II-e), according to the phylogenetic analysis of the WRKY domains. Group III WRKY TFs also contain a single WRKY domain but differ from Group I and II in their altered C_2_HC-type of zinc-finger motif [5,6]. Minor variations in the WRKYGQK sequence motif such as WRKYGEK, WRKYGKK, WRKYGSK, and WRKYDQK were also reported [7,8,9].

The WRKY family was widely studied in *Arabidopsis thaliana* and *Oryza* *sativa* for years, where many were related to phytohormones, and abiotic and biotic stresses [10,11]. In addition, several WRKY members of this family were found to have regulatory functions in many plant developmental processes such as trichome and seed coat initiation, germination, seed dormancy, leaf senescence, and secondary metabolism [12,13,14,15,16]. A principal elicitor of triterpene ginsenoside biosynthesis in *Panax* species and natural product biosynthesis in a number of medicinal plants is MeJA, which plays a pivotal role in plant defense signaling in response to biotic stresses [17,18]. Recently, over-expression of MeJA-responsive *PqWRKY1* in *Arabidopsis* has been shown to enhance biotic and abiotic stress tolerance, in addition to the gene expression related to ginsenoside biosynthesis [19]. More recently, a *PgWRKY1* gene in *P. ginseng* has been identified responding to a salt (NaCl) and various hormones (MeJA, SA, and ABA) [8].

Ginseng (*Panax ginseng* C.A. Meyer), known as the king of herbs, has numerous pharmaceutical effects associated with several types of diseases such as obesity, cancer, stress, diabetes, cardiovascular, and aging-related diseases [20,21,22,23,24]. Triterpene ginsenoside is the main pharmacologically active compound in *P. ginseng* affecting the value of the herb [25,26,27]. Although *WRKY* genes are important in plant growth and development, only two *WRKY* genes (*PqWRKY1* and *PgWRKY1*) from different *Panax* species have been characterized to date [8,19]. In our lab, the study was initiated with the objective of transcriptome-wide identification of *WRKY* unigenes, where one (*PgWRKY1*) out of 48 candidates that seemed to respond to a salt and various hormones was cloned [8]. Thus, WRKY TFs were thought to be involved in various stress responses in this species. In this study, we therefore further cloned a number of *PgWRKY* genes encoding PgWRKY proteins belonging to three subgroups (one subgroup II-c, four subgroup II-d, and three subgroup II-e). Moreover, their expression patterns in different tissues and expression profiles under various hormones and NaCl stress conditions were performed using qRT-PCR analysis. The results will provide valuable information for other WRKY groups to dissect further molecular mechanisms and their functionalities.

## 2. Results

### 2.1. Cloning and Phylogeny

**Figure 1:**
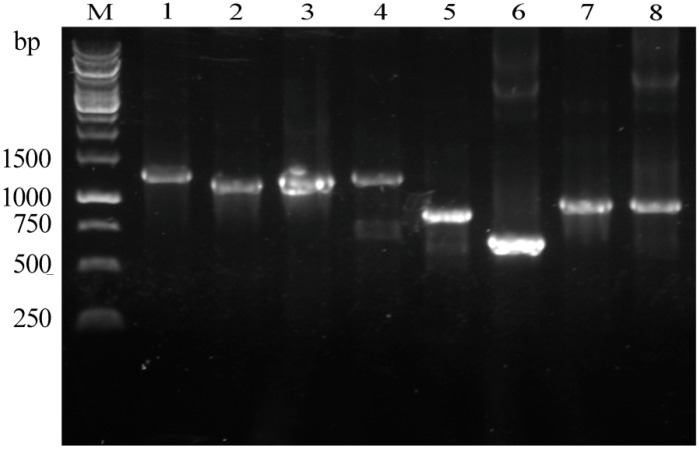
Full-length cDNAs of the eight studied *PgWRKYs* (*PgWRKY2-9*). M: Marker (1 kbp DNA Ladder); Lane 1: *PgWRKY2* (1261 bp); Lane 2: *PgWRKY3* (1196 bp); Lane 3: *PgWRKY4* (1233 bp); Lane 4: *PgWRKY5* (1259 bp); Lane 5: *PgWRKY6* (890 bp); Lane 6: *PgWRKY7* (700 bp); Lane 7: *PgWRKY8* (1071 bp); and Lane 8: *PgWRKY9* (987 bp).

Through deep mining of our MeJA-treated *P. ginseng* transcriptome dataset, eight annotated *PgWRKY* unigenes (CL196. Contig1, CL495. Contig1, CL495. Contig8, CL196. Contig2, CL4686. Contig3, CL1589.Contig3, CL5364. Contig3, and Unigene 6844) were selected for cloning, showing obvious regulation in the dataset [8]. The primers were designed then and the selected candidates designated as *PgWRKY2*, *PgWRKY3*, *PgWRKY4*, *PgWRKY5*, *PgWRKY6*, *PgWRKY7*, *PgWRKY8*, and *PgWRKY9* (GenBank accession number: KR060075-KR060079 and KU144580-KU144582) were successfully cloned. Sequence analysis showed that the eight full-length cDNAs ranged from 700 (*PgWRKY7*) to 1261 (*PgWRKY2*) bp (Figure 1, Figure S1). The length of the open reading frames (ORFs) of the *PgWRKY* genes vary from 585 (*PgWRKY7*) to 1077 bp (*PgWRKY3/4*), encoding proteins of 194–358 amino acids. Their molecular weight (*M*_W_) comprised from 21.59 to 39.80 kDa and their iso-electric point (PI) from 5.03 to 9.56.

**Figure 2:**
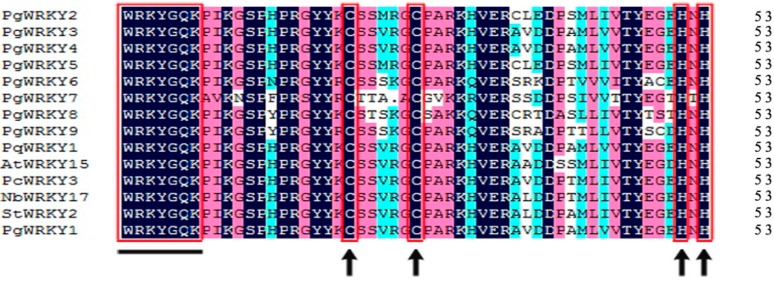
WRKY domain comparison of the eight analyzed PgWRKY TFs (PgWRKY2-9) with other members of WRKY Group II. Amino acid sequence analysis of the eight PgWRKYs with *P. quinquefolius* PqWRKY1 (AEQ29014.1), *A. thaliana* AtWRKY15 (AEC07442.1), *Petroselinum crispum* PcWRKY3 (AAC49528.1), *Nicotiana benthamiana* NbWRKY17 (AIR74899.1), *Solanum tuberosum* StWRKY2 (NP_001275001.1), and *P. ginseng* PgWRKY1 (KR060074) was accomplished using DNAMAN software. The WRKYGQK sequence motifs are underlined and cysteines (C) and histidines (H) in the zinc-finger motifs are indicated by arrowheads.

**Figure 3:**
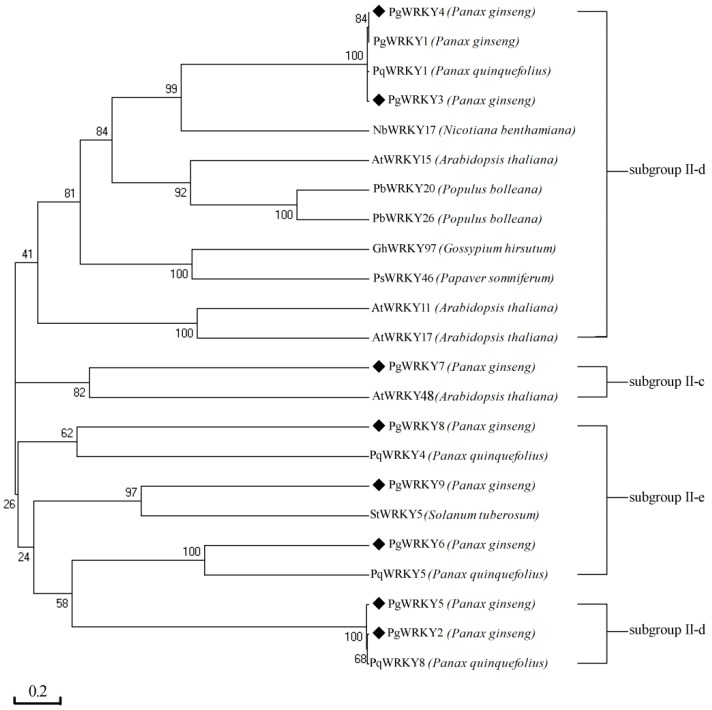
Phylogenetic relationships among the eight studied proteins (PgWRKY2-9) and other members of the WRKY family estimated based on their amino acid sequences. WRKY amino acid sequences of the following proteins were used to make the tree: PqWRKY1 (AEQ29014.1), PgWRKY1 (KR060075), NbWRKY17 (AIR74899.1), AtWRKY15 (NP_179913.1), PbWRKY20 (ACV92022.1), PbWRKY26 (ACV92028.1), GhWRKY97 (AGV75972.1), PsWRKY46 (AFU81789.1), AtWRKY11 (NP_849559.1), AtWRKY17 (NP_565574.1), AtWRKY33 (NM_129404.3), PqWRKY4 (AEQ29017.1), StWRKY5 (NP_001274847.1), PqWRKY5 (AEQ29018.1), and PqWRKY8 (AEQ29021.1). The neighbor-joining phylogenetic tree was constructed using the bootstrap method of MEGA 4.0 with 1000 replications and the respective plant species of the above proteins are shown in the tree. The black square symbols indicate the eight studied PgWRKY proteins (PgWRKY2-9).

In comparison with the sequence alignment of Group II WRKY proteins from different plants, each of the eight studied PgWRKY proteins was found to have a single WRKY domain consisting of one WRKYGQK sequence motif located at the N-terminal and one C_2_H_2_-type zinc-finger motif at the C-terminal (Figure 2). Therefore, these PgWRKY TFs were assigned to Group II according to the WRKY classification criteria [3,5]. Moreover, in Group II, the four PgWRKYs were assigned to subgroup II-d (PgWRKY2, PgWRKY3, PgWRKY4, and PgWRKY5), one to subgroup II-c (PgWRKY7), and three to subgroup II-e (PgWRKY6, PgWRKY8, and PgWRKY9) as per the corresponding unigenes of our transcriptomic dataset [8] and phylogenetic tree (Figure 3). Four PgWRKY proteins were found to be highly similar to PgWRKY1, PqWRKY1, StWRKY5, and PqWRKY5 and four PgWRKYs were found to be similar to PgWRKY1, AtWRKY48, PqWRKY4, and PqWRKY8. These observations point out that the eight *PgWRKYs* are members of the *WRKY* gene family.

### 2.2. Expression Pattern of PgWRKY Genes in Different Tissues

**Figure 4:**
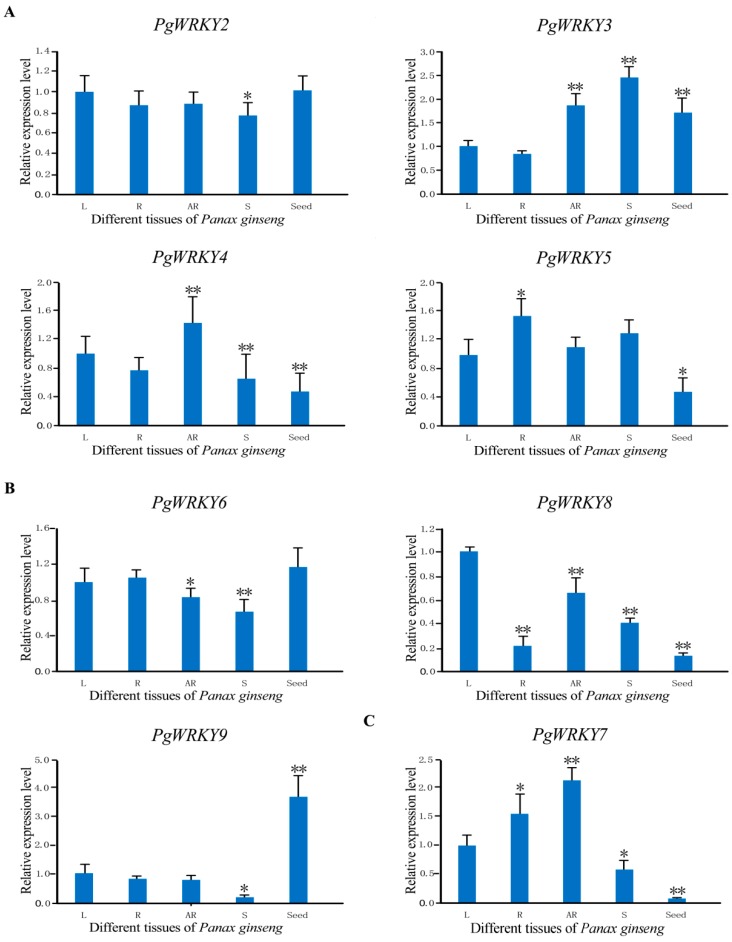
Tissue-specific expression of the eight studied *PgWRKYs* (*PgWRKY2-9*) in different tissues of *P. ginseng* analyzed by qRT-PCR. (**A**) Subgroup II-d; (**B**) Subgroup II-e; and (**C**) Subgroup II-c. L: Leaves, R: Roots, AR: Adventitious roots, and S: Stems. Error bars represent standard deviation (SD) of means from three independent experiments. The relative expression was calculated using the 2^−∆∆*C*t^ method based on the corresponding gene expression in leaves. The differences between the transcript level of adventitious roots, roots, leaves, stems, and seeds are statistically significant (* p < 0.05, ** p < 0.01) according to independent samples *t*-test.

Emerging evidence indicated that *WRKY* genes show various expression patterns in different plant tissues. For example, *CrWRKY1* in *Catharanthus* *roseus* was found to preferentially be expressed in roots [28], and *OsWRKY82* was shown to differentially be expressed in all tested organs including stems, leaves, grains, and flowers [29]. In this study, the expression patterns of the eight studied *PgWRKY* genes were investigated in different *P. ginseng* tissues using qRT-PCR analysis (Figure 4). The results revealed that the genes were expressed differentially in leaves, roots, adventitious roots, stems, and seeds. In subgroup II-d, the expression level of the four surveyed *PgWRKY* genes showed functional diversity, with a relative increased expression level of *PgWRKY3* in stems (2.44-fold), *PgWRKY4* in adventitious roots (1.43-fold), and *PgWRKY5* in roots (1.54-fold), compared with that in leaves (one-fold). In subgroup II-e, *PgWRKY9* expression was the highest in seeds (3.67-fold). However, the expression of *PgWRKY8* assigned to the same group was relatively lower in all the investigated organs compared with that in leaves. The remaining *PgWRKY7* belonging to subgroup II-c was expressed at the highest level in adventitious roots (2.12-fold). We further observed the decreased transcripts of the eight *PgWRKY* genes in the tested organs compared to leaves. The lowest expression levels were shown by the following genes: *PgWRKY2*, *PgWRKY6*, and *PgWRKY9* in stems (0.77-, 0.68-, and 0.19-fold), *PgWRKY3* in roots (0.84-fold), and *PgWRKY7* and *PgWRKY8* in seeds (0.05- and 0.13-fold). Likewise, the expression levels of *PgWRKY4* and *PgWRKY5* were observed at the same lowest level in seeds (0.47-fold). The results imply that the *WRKY* members belonging to different subgroups might have diverse functions.

### 2.3. Expression Analysis of PgWRKY Genes under Different Treatments

**Figure 5:**
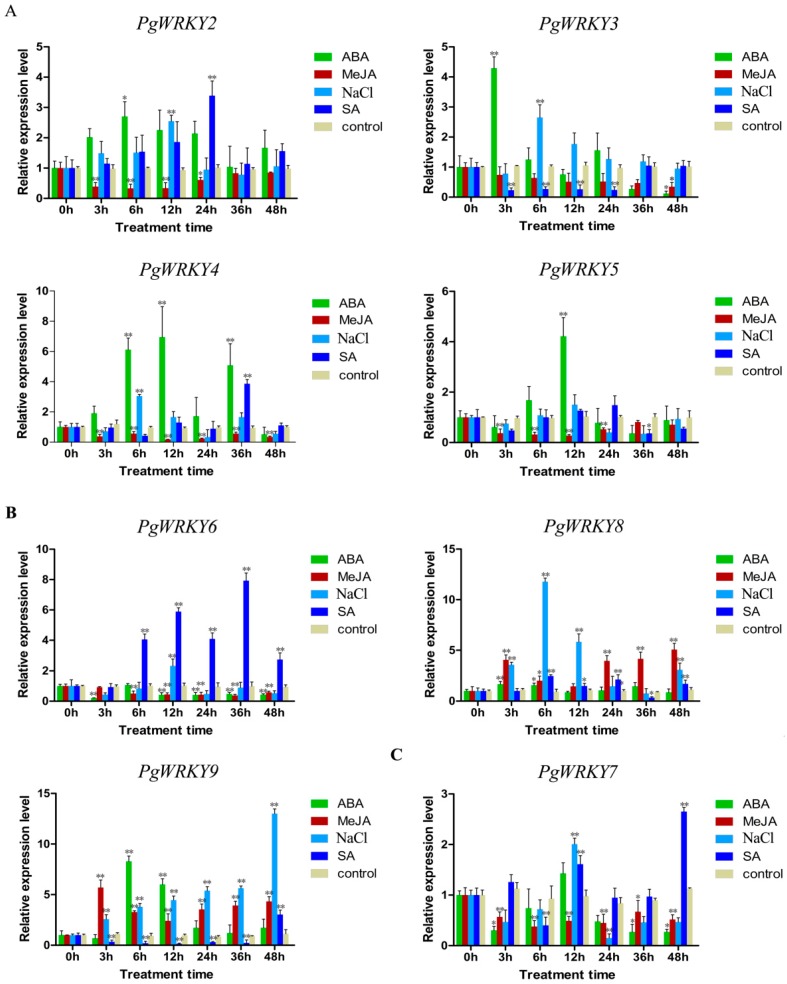
Expression profiles of the eight analyzed *PgWRKYs* (*PgWRKY2-9*) in *P. ginseng* hairy roots treated with 200 µM MeJA, 200 µM SA, 20 µM ABA, and 50 mM NaCl. (**A**) Subgroup II-d; (**B**) Subgroup II-e; and (**C**) Subgroup II-c. The hairy roots treated with equal amount of solvent of the corresponding chemicals were used as control (0, 3, 6, 12, 24, 36, and 48 h). The transcript levels at different time points (0, 3, 6, 12, 24, 36, and 48 h) were measured by qRT-PCR. The levels of gene expression in the treated samples were analyzed on the basis of the corresponding gene expression at 0 h using the 2^−∆∆*C*t^ method. The error bars represent the standard deviation (SD) from triplicate experiments. The differences between the level of transcript of treated and control hairy roots at 0 h are statistically significant (* p < 0.05) and extremely significant (** p < 0.01) according to independent samples *t*-test.

To investigate which *PgWRKY* genes were significantly regulated in *P. ginseng* hairy roots, we performed qRT-PCR analysis at different time points following various treatments: MeJA, SA, ABA, and NaCl (Figure 5). The results showed that six *PgWRKY* genes were significantly downregulated in hairy roots treated with MeJA, with four in subgroup II-d (*PgWRKY2*, *PgWRKY3*, *PgWRKY4*, and *PgWRKY5*), one in subgroup II-e (*PgWRKY6*), and one in subgroup II-c (*PgWRKY7*). Among them, *PgWRKY4*, *PgWRKY6*, and *PgWRKY7* showed reduced expression at all time points of treatment compared to the control. The remaining two *PgWRKYs* (*PgWRKY8* and *PgWRKY9*) belonging to subgroup II-e were found to be induced during all time points of MeJA treatment except in the 12 h treatment of *PgWRKY8*. The statistical analysis showed that the changes in the expression levels of *PgWRKYs* with MeJA were significant (*p* < 0.05 or < 0.01). Under SA treatment, the transcripts of three *PgWRKY* genes (*PgWRKY3*, *PgWRKY5*, and *PgWRKY9*) were found to decrease significantly. The expression level of *PgWRKY3* decreased until 24 h and returned to the control level thereafter. The *PgWRKY5* expression was found to be downregulated only at 36 h, whereas the *PgWRKY9* was reduced until 36 h post-treatment. The *PgWRKY2* was significantly induced only at 24 h and *PgWRKY4* at 36 h. The transcripts of *PgWRKY6* were accumulated quickly from 6 to 48 h compared to the control. The expression level of *PgWRKY7* significantly decreased at 6 h but increased at 12 h and its highest level of expression was found at 48 h post-treatment. The transcripts of *PgWRKY8* started to significantly increase from 6 h and this trend was observed till 48 h with the exception of 36 h of SA treatment. During ABA treatment, the transcripts of five *PgWRKYs* (*PgWRKY2*, *PgWRKY4*, *PgWRKY5*, *PgWRKY8*, and *PgWRKY9*) were found to be significantly upregulated in comparison with the control. The transcripts of *PgWRKY2* were induced only during 6 h and *PgWRKY5* only during 12 h of treatment. The level of *PgWRKY8* expression increased slightly from 3 to 6 h compared to the control, while a remarkable increase in the transcripts of *PgWRKY9* was observed from 6 to 12 h. The transcripts of *PgWRKY4* were found to be accumulated at three time points of treatments including 6, 12, and 36 h. The expression level of *PgWRKY3* rapidly increased at the beginning of the treatment but decreased dramatically at 48 h, while the decreased transcripts of *PgWRKY6* were obtained at the beginning and this trend lasted from 12 to 48 h. Likewise, the expression level of *PgWRKY7* declined at the start of ABA treatment and the reduction was also observed between 36 and 48 h of ABA treatment. In NaCl treatment, the transcript levels of all the tested *PgWRKYs* were significantly upregulated or downregulated, except *PgWRKY5*. Among them, only the transcripts of *PgWRKY9* were induced at all time points of treatment in comparison with the control. It was found to increase rapidly at the beginning of NaCl treatment and continually increased with the passage of time until the highest level was reached at 48 h (13.01-fold). Moreover, the induced expression of *PgWRKY2* and *PgWRKY6* was observed only at 12 h, while *PgWRKY3* and *PgWRKY4* showed this trend during 6 h of the salt treatment. The expression level of *PgWRKY7* also increased at 12 h, but declined when it was treated for the period of 24 h. The transcript level of *PgWRKY8* was found to significantly increase at four time points (3, 6, 12, and 48 h) of NaCl treatment. The statistical analysis indicated that the changes in the expression levels of all the analyzed *PgWRKYs* except *PgWRKY5* were extremely significant (*p* < 0.01) upon NaCl treatment.

Integrating the gene expression results of the eight *PgWRKYs* during four different treatments (Table 1), the transcripts of the *PgWRKY9* belonging to subgroup II-e accumulated simultaneously at all time points of MeJA and NaCl treatments with the highest peak at 3 h (5.71-fold) and 48 h (13.01-fold), respectively. The expression of *PgWRKY2* belonging to subgroup II-d was induced by both SA and ABA with the best induced expression during 24 h (3.39-fold) of SA treatment. Moreover, among all the treatments, the highest induced expression of *PgWRKY3*, *PgWRKY4*, and *PgWRKY5* was observed with the treatment of ABA including 4.29-fold at 3 h, 6.95-fold at 12 h, and 4.22-fold at 12 h, respectively, in comparison with the control. Likewise, *PgWRKY6* transcripts showed the highest induced expression with SA (7.93-fold at 36 h), *PgWRKY7* also with SA (2.65-fold at 48 h), and *PgWRKY8* with NaCl (11.78-fold at 6 h). However, only the *PgWRKY5* belonging to the set of eight *PgWRKY* genes did not show any significant regulation upon NaCl treatment. Interestingly, MeJA did not induce *PgWRKY2/3/4/5/6/7* expression; it was instead significantly reduced by MeJA. The lowest accumulation of *PgWRKY2* transcripts was observed at 6 h (0.33-fold), *PgWRKY4* at 12 h (0.17-fold), and *PgWRKY5* also at 12 h (0.27-fold) of MeJA treatment. Moreover, *PgWRKY3* and *PgWRKY6* showed the lowest expression at 48 h (0.12-fold) and at 3 h (0.20-fold) of ABA treatment, respectively. Likewise, the *PgWRKY7* transcripts were accumulated at the lowest level at 24 h (0.15-fold) of NaCl treatment. In SA treatment, the expression level of *PgWRKY8* was found to be the lowest at 36 h (0.35-fold) and *PgWRKY9* at 12 h (0.14-fold). These observations indicate that *PgWRKY* genes are multiple stress–inducible genes responding to NaCl and various hormones.

## 3. Discussion

WRKY proteins in higher plants play important roles in response to various physiological processes, particularly in biotic and abiotic stresses [4,30]. In recent years, many *WRKY* genes have been isolated and functionally characterized, mostly in model plants such as *Arabidopsis* and rice [5,31,32]. Moreover, a number of *WRKY* genes and their roles have been identified in many medicinal plants including *Coptis* *japonica*, *Catharanthus* *roseus*, *Artemisia* *annua*, and *P.* *quinquefolius* [15,19,28,33]. In spite of the pharmaceutical and industrial importance related to *P. ginseng* from ancient times, only one *WRKY* gene (*PgWRKY1*) has previously been characterized in *P. ginseng* [8]. In this study, the *PgWRKY2/3/4/5/6/7/8/9* genes cloned and characterized will provide more insight into expression profiles under MeJA, SA, ABA, and salt treatments. To elucidate the relationships among the WRKY DNA binding regions from different plants, we carried out multiple alignment analysis (Figure 2) which shows high sequence identities of PgWRKY2/3/4/5/6/7/8/9 proteins, indicating that amino acid residues are highly conserved among WRKY proteins. Phylogenetic tree analysis (Figure 3) showed that PgWRKY2/3/4/5/6/7/8/9 and other plant WRKYs are homologous, suggesting a probable functional similarity among them. Expression analysis of *PgWRKY2/3/4/5/6/7/8/9* in various organs (Figure 4) revealed that *PgWRKY5* was relatively higher in roots, *PgWRKY3* in stems, *PgWRKY4* and *PgWRKY7* in adventitious roots, and *PgWRKY9* in seeds compared with leaves, suggesting that they might be involved in the development of all *P. ginseng* organs.

The *WRKY* genes were previously identified to respond to different hormones and abiotic stresses and to be involved in a number of life processes including stress resistance, defense, and secondary metabolism [15,34,35,36]. About 109 *OsWRKY* genes were identified in rice [10,29,37], and mostly were shown to be involved in many hormones, biotic stress, and abiotic stress responses. Among various elicitors, MeJA was identified to be an effective elicitor for the induction of ginsenoside contents in hairy roots and cultured cells of *Panax* species [18,38]. The recently discovered *PqWRKY1* in *P. quinquefolius* related to triterpene ginsenoside biosynthesis was induced rapidly upon MeJA treatment [19]. More recently, we identified the *PgWRKY1* in *P. ginseng* to be downregulated by MeJA, but upregulated upon SA, ABA, and salt treatments [8]. Therefore, the exploration of the functions of various treatments and salt-responsive *WRKY* genes in *P. ginseng* will be helpful in discovering genes involved in stress resistance and secondary metabolite biosynthesis. In the present study, we further identified six *PgWRKY* genes (*PgWRKY2*, *PgWRKY3*, *PgWRKY4*, *PgWRKY5*, *PgWRKY6*, and *PgWRKY7*) to significantly be downregulated at all time points of MeJA treatment, whereas two *PgWRKYs* (*PgWRKY8* and *PgWRKY9*) were found to be induced during this treatment (Figure 5). MeJA-regulated *WRKY* genes were previously identified to be regulated by SA, ABA, and salt [39,40]. For example, the transcripts of *LtWRKY21* in *Larrea tridentate* vegetative tissues were induced by ABA and in response to various environmental stresses such as water deficit, wounding, drought, and salt stresses [41]. Moreover, the major part of *Arabidopsis* *WRKY* TF-related genes was shown to be induced by SA treatment [11,42]. We therefore tried to observe if the expression profiles of the eight studied *PgWRKYs* were related to SA, ABA, and salt stresses, where all the analyzed genes were found to differentially be expressed with these treatments (Figure 5). SA treatment led the best induced expression of *PgWRKY6* (7.93-fold). Likewise, most of these *PgWRKY* genes were also found to significantly increase with the treatment of ABA and NaCl. Among the eight *PgWRKY* genes, the transcripts of the *PgWRKY9* were found to have maximum induction with the treatment of ABA and NaCl with 8.28- and 13.01-fold, respectively. These findings indicated that *PgWRKY* genes have multi-functional roles in various hormone and salt treatments and may potentially be applied in breeding new *P. ginseng* cultivars with increased stress resistance.

## 4. Materials and Methods

### 4.1. Plant Materials

Commercial cultivars of ginseng (*Panax* *ginseng* C.A. Meyer) with four-year-old were collected from the forest of the Changbai mountain of Fusong County, Baishan, Jilin Province, China. Untreated fresh hairy roots were used to extract RNA for gene cloning and treated hairy roots with different hormones (MeJA, SA, and ABA) and a salt (NaCl) were used to observe expression profiles. Ginseng roots, adventitious roots, stems, seeds, and leaves were used separately for RNA isolation and tissue-specific expression analysis.

### 4.2. Cloning of PgWRKYs

Based on the selected eight *PgWRKYs* unigenes from our previous *P. ginseng* tanscriptome dataset [8], gene-specific primers (Table 2) were individually designed for PCR amplification of the *PgWRKY* genes based on the complete ORF cDNA sequences. Total RNA was extracted from hairy roots by E.Z.N.A plant RNA Kit (Omega Bio-Tek, Doraville, GA, USA) according to the manufacturer’s protocols and then treated by RNase-free DNase I (Omega Bio-Tek) to prevent residual DNA. The first-strand cDNA was synthesized in a reaction volume of 20 µL using 1 µg of total RNA and RevertAid First-Strand cDNA synthesis Kit (Thermo Fisher Scientific, Waltham, MA, USA) following the manufacture’s procedure. PCR reaction was carried out in a 16 µL reaction volume including 8 µL of 2× Taq MasterMix, 1 µL of each primer (10 µM), 5.5 µL of ddH_2_O, and 0.5 µL of cDNA template. The PCR conditions were as follows: 94 °C for 1 min; 94 °C for 30 s, 50–58 °C for 30 s (*PgWRKY3* and *PgWRKY6*: 50 °C, *PgWRKY7*, *PgWRKY8*, and *PgWRKY9*: 52 °C, and *PgWRKY2*, *PgWRKY4*, and *PgWRKY5*: 58 °C), 72 °C for 1 min with 35 cycles; and 72 °C for 10 min. All of the amplified PCR products were purified using Wizard SV Gel and PCR Clean-Up System Kit (Promega, Madison, WI, USA). We then cloned the purified PCR fragments into the pGEM-T Easy vector (Promega) (Figure S2) and subsequently sequenced the successful clones by Invitrogen (Pudong, Shanghai, China). To calculate the theoretical PI and MW of the studied PgWRKY proteins, their respective amino acid sequences were pasted in the box found using ExPASy server (http://web.expasy.org/protparam/). The amino acid sequences of PgWRKY proteins and other plant WRKY proteins were aligned using CLUSTAL_X and the phylogenetic tree was generated with the MEGA4.0 program using NJ algorithm with 1000 bootstrap trials [43]. Multiple sequence alignments using amino acid sequences of the studied PgWRKY proteins were determined with DNAMAN software (http://www.lynnon.com/alignm.html). Primers were designed with primer premier 5.0 software (http://www.idtdna.com/Primerquest/Home/Index).

### 4.3. Salts and Hormone Treatments

To analyze the responses of *PgWRKY* genes to a salt and various hormone treatments, hairy roots cultured for two weeks were transferred to eight conical flasks, each of which contained 40 mL Murashige and Skoog (MS) medium. Subsequently, 200 µM MeJA, 200 µM SA, 20 µM ABA, and 50 mM NaCl were individually added in four of the flasks. Equal amount of solvents used to dissolve the hormones and salt were added in the remaining four flasks (empty control). All the flasks were stored on a rotary shaker with 180 rpm and 28 °C in dark. The hairy root samples following 0, 3, 6, 12, 24, 36, and 48 h treatment were collected immediately and frozen in liquid nitrogen and then stored at −80 °C for RNA extraction.

### 4.4. Expression Analysis

The transcript levels of *PgWRKY* genes from various organs and hairy roots of *P. ginseng* were measured by qRT-PCR with UltraSYBR mixture (with ROXII) (CWBIO, Beijing, China) using the Mastercycler ep realplex^2^ detection system (Eppendorf, Humberg, Germany). Total RNA using E.Z.N.A plant RNA Kit was isolated from the salt and hormone treated hairy root samples and then reversely transcribed into cDNAs using RevertAid First-Strand cDNA Synthesis Kit (Fermentas, Waltham, MA, USA). Gene-specific primers and *Pgβ-actin* (internal control) primers (Table 3) were used for qRT-PCR analysis. The amplification of cDNA was performed in a 50 µL reaction mixture containing 25 µL of 2× UltraSYBR Mixture (with ROXII), 1 µL of each 10 µM primers (forward and reverse), 50 ng of cDNA, and ddH_2_O up to 50 µL. The PCR conditions were as follows: 95 °C for 10 min; 95 °C for 30 s, 60–62 °C for 30 s (*PgWRKY7*, *PgWRKY8*, and *PgWRKY9*: 60 °C, and *PgWRKY2*, *PgWRKY3*, *PgWRKY4*, *PgWRKY5*, and *PgWRKY6*: 62 °C), 72 °C for 1 min, 40 cycles. A melting curve analysis was performed following amplification cycles to validate the specificity of the reactions. This was carried out by heating all the amplified fragments between 60 and 95 °C at 0.5 °C per 10 s. Post qRT-PCR calculations were performed to analyze relative gene expression levels of the studied *PgWRKY* genes based on the 2^−∆∆*C*t^ method as explained earlier [44]. The results obtained for the level of gene expression were demonstrated following normalization with the *Pgβ-actin* used as internal control. All the qRT-PCR amplifications were repeated three times.

### 4.5. Statistical Analysis

Data from qRT-PCR were presented as the mean ± standard deviation (SD) of three independent experiments, each of those was repeated three times. Analysis of variance (ANOVA) was used to conduct statistical analysis and comparison of means was performed using the least significant difference (LSD) test at *p* = 0.05. The differences were considered statistically significant when *p* < 0.05 and extremely significant when *p* < 0.01.

## 5. Conclusions

In this study, we cloned eight *PgWRKY* cDNAs from *P. ginseng* along with their expression patterns in different tissues and expression profiles under a salt and various hormones. We found two *PgWRKYs* to be upregulated while others were downregulated following MeJA treatments. Furthermore, the genes were regulated differentially at transcript levels by SA, ABA, and salt treatments. The observations of the analyzed *PgWRKYs* will provide a comprehensive overview of the WRKY family in *P. ginseng* which might contribute in guiding ginseng breeding and discovering genes related to stress tolerance and secondary metabolite biosynthesis in *Panax* *ginseng* and related species.

## Figures and Tables

**Table 1:** Expression profiles of the eight analyzed *PgWRKYs* (*PgWRKY2-9*) under various treatments.

Gene	Group	MeJA	SA	ABA	NaCl
*PgWRKY2*	Group II-d	D	**	*	**
*PgWRKY3*	Group II-d	D	D	•	**
*PgWRKY4*	Group II-d	D	**	**	**
*PgWRKY5*	Group II-d	D	D	**	-
*PgWRKY6*	Group II-e	D	**	D	**
*PgWRKY7*	Group II-c	D	•	D	•
*PgWRKY8*	Group II-e	**	•	**	**
*PgWRKY9*	Group II-e	**	•	**	**

Treatments of hairy roots with 200 µM MeJA, 200 µM SA, 20 µM ABA, and 50 mM NaCl were performed for 0, 3, 6, 12, 24, 36, and 48 h. D: Significant downregulation of *PgWRKY* genes expression at *p* < 0.05 or 0.01; ** & *: Significant upregulation of *PgWRKY* genes at *p* < 0.01 & *p* < 0.05 respectively; •: Significant differential regulation of *PgWRKY* genes at *p* < 0.05 or *p* < 0.01; -: *PgWRKY* genes were not significantly regulated.

**Table 2:** List of primers used for cloning of eight *PgWRKYs* (*PgWRKY2-9*).

Primer Name	Product Length (bp)	Primer Sequence (5′ to 3′)
PgWRKY2-F	1261	TCTTTTGGGTGTGTGAAT
PgWRKY2-R		TTTGGCGTTTGTATGGTA
PgWRKY3-F	1196	CTTTGCTTTTCCTTAGTTG
PgWRKY3-R		ATCATTATCTCTCTCCTTGG
PgWRKY4-F	1233	CTTTGCTTTTCCTTAGTTG
PgWRKY4-R		AACAAACATTTCCAACCG
PgWRKY5-F	1259	TCTTTTGGGTGTGTGAAT
PgWRKY5-R		TGGCGTTTGTATGGTAAT
PgWRKY6-F	890	CTTACCAGCCCTCTCCG
PgWRKY6-R		ATACTTTTGTAAGGAGGACTA
PgWRKY7-F	700	AAACGCTAAAACCCAAGA
PgWRKY7-R		ATTTTCTCTTGCTGACTTCT
PgWRKY8-F	1071	TGTGTGGGTTTGTCGTCT
PgWRKY8-R		CGCTACTATTAAATCTTGGC
PgWRKY9-F	987	TCTCTCCTCCATAACAACT
PgWRKY9-R		AAAAAAAGACAAATCCCC

**Table 3:** List of primers of eight *PgWRKYs* (*PgWRKY2-9*) and *Pgβ-actin* used for qRT-PCR analysis.

Primer Name	Product Length (bp)	Primer Sequence (5′ to 3′)
PgWRKY2-F	115	TTTATCTCCTCGTTGAGTGTCG
PgWRKY2-R		ACCTTCGTTTGTGCTGGTATG
PgWRKY3-F	110	CGGAAACCCAGACTCACG
PgWRKY3-R		TCGGGACCCATTAGACATCA
PgWRKY4-F	134	CACCAACAGCATCAACAGCG
PgWRKY4-R		ATTCGGGACCCATTAGACATCA
PgWRKY5-F	147	GGAGCAATAGTGGCATCAACCTG
PgWRKY5-R		GAGTCGCACCAATTAAATGGAAAG
PgWRKY6-F	84	CCATTCCCGTCAAGTTTCCAC
PgWRKY6-R		CGGCAAAGTCTATGTTGTCAGG
PgWRKY7-F	169	CGACATTATCTTCCTTCAACTTTA
PgWRKY7-R		ACTCGCATTTGGGACGGC
PgWRKY8-F	127	ATGGATAACTCTCCCTCCCCTA
PgWRKY8-R		CATTCTCTTCAATCTTCACTGC
PgWRKY9-F	118	GCAGAAAAGAGTGGTGTCCG
PgWRKY9-R		AGGCTTTTGACCATACTTTC
Pgβ-Actin-F	176	GCGGTTGAGGTGGTGGGT
Pgβ-Actin-R		GTCCTACTAACAAGGCAGAG

## Supplementary Materials

Supplementary materials can be found at http://www.mdpi.com/1422-0067/17/3/319/s1.
