# Supplementary material for: Molecular Cloning and Expression Analysis of Eight PgWRKY Genes in Panax ginseng Responsive to Salt and Hormones
Source: Int J Mol Sci. 2016 Mar 4;17(3):319. doi: 10.3390/ijms17030319 (PMC4813182; doi:10.3390/ijms17030319)
Supplement: Supplementary file 1 [file ijms-17-00319-s001.pdf]

Supplementary Materials: Molecular Cloning and Expression Analysis of Eight *PgWRKY* Genes in *Panax ginseng* Responsive to Salt and Hormones

Hao Xiu, Mohammed Nuruzzaman, Xiangqian Guo, Hongzhe Cao, Jingjia Huang, Xianghui Chen, Kunlu Wu, Ru Zhang, Yuzhao Huang, Junli Luo and Zhiyong Luo

PgWRKY5

1	ATG	GAG	GTT	GAA	GAA	GCT	AAC	AAA	GCA	GCA	GTT	GAG	AGT	TGC	CAC	45
1	M	E	V	E	E	A	N	K	A	A	V	E	S	C	H	15
46	AGA	GTT	CTA	AGT	CTG	ATA	TCT	CAG	CCC	CAA	GAT	CAG	ATT	CAG	TAT	90
16	R	V	L	S	L	I	S	Q	P	Q	D	Q	I	Q	Y	30
91	AGG	AAT	TTA	GTG	GTT	GAA	ACT	GGG	GAA	GCT	GTA	CTT	AAG	TTT	GAG	135
31	R	N	L	V	V	E	T	G	E	A	V	L	K	F	E	45
136	AAA	GTT	GTT	TCT	CTT	CTT	AAT	GCT	GGT	TTG	GGG	CAT	GCA	AGA	GTG	180
46	K	V	V	S	L	L	N	A	G	L	G	H	A	R	V	60
181	AGA	AAG	ATC	GAG	AAA	ATT	CAA	ACC	CCT	TTT	CCC	CAA	AAC	ATA	CTC	225
61	R	K	I	E	K	I	Q	T	P	F	P	Q	N	I	L	75
226	TTA	GAC	AAC	CCA	ATT	GGT	AGA	CCT	GAT	TAT	CAA	CCG	AAA	GCG	ATT	270
76	L	D	N	P	I	G	R	P	D	Y	Q	P	K	A	I	90
271	CAG	CTT	CTT	CCA	GCT	AAT	TCC	CTT	GAG	ACC	CCA	ATT	CAT	GAT	AAG	315
91	Q	L	L	P	A	N	S	L	E	T	P	I	H	D	K	105
316	GGT	TCA	AAT	GTT	CGA	AGT	ACT	CTT	ACT	TTA	GGA	AAT	TCA	TCA	TTG	360
106	G	S	N	V	R	S	T	L	T	L	G	N	S	S	L	120
361	GAA	CTG	AGT	TCA	AAT	GGG	AAA	AAC	TCT	CTT	CAA	ATA	CCC	CAA	CAA	405
121	E	L	S	S	N	G	K	N	S	L	Q	I	P	Q	Q	135
406	ACA	CCA	CCA	TCA	AAC	TAT	CAC	TTC	CTG	CAG	CAA	AAG	TTT	CAG	CTT	450
136	T	P	P	S	N	Y	H	F	L	Q	Q	K	F	Q	L	150
451	CAG	CAG	CAA	CAG	TTA	AAA	CAG	CAA	ACT	GAA	ATG	ATG	TTC	CGT	CGG	495
151	Q	Q	Q	Q	L	K	Q	Q	T	E	M	M	F	R	R	165
496	AGC	AAT	AGT	GGC	ATC	AAC	CTG	AAT	TTT	GAT	AGC	TCT	ACC	TGC	ACG	540
166	S	N	S	G	I	N	L	N	F	D	S	S	T	C	T	180
541	CCC	ACC	ATG	TCA	TCC	ACT	AGG	TCA	TTT	ATC	TCC	TCA	TTG	AGT	GTC	585
181	P	T	M	S	S	T	R	S	F	I	S	S	L	S	V	195
586	GAT	GGG	AGT	GTG	GCT	AAT	ATG	GAG	GGC	AAT	GCT	TTC	CAT	TTA	ATT	630
196	D	G	S	V	A	N	M	E	G	N	A	F	H	L	I	210
631	GGT	GCG	ACT	CGC	TCT	TTG	GAT	CAG	AGC	TCA	TAC	CAG	CAC	AAA	CGA	675
211	G	A	T	R	S	L	D	Q	S	S	Y	Q	H	K	R	225
676	AGG	TGC	TCT	GCA	AAG	GGA	GAT	GAT	GGC	AGT	GTG	AAA	TGT	GGA	AGC	720
226	R	C	S	A	K	G	D	D	G	S	V	K	C	G	S	240
721	AGT	GGT	AGA	TGT	CAC	TGC	TCA	AAG	AAG	AGG	AAA	CAT	AGG	GTA	AAG	765
241	S	G	R	C	H	C	S	K	K	R	K	H	R	V	K	255
766	AGA	TCA	ATC	AAA	GTA	CCT	GCT	ATA	AGC	AAC	AAG	CTC	GCA	GAC	ATC	810
256	R	S	I	K	V	P	A	I	S	N	K	L	A	D	I	270
811	CCT	CCT	GAT	GAG	TAT	TCA	TGG	AGG	AAG	TAT	GGT	CAG	AAA	CCA	ATC	855
271	P	P	D	E	Y	S	W	R	K	Y	G	Q	K	P	I	285
856	AAG	GGT	TCT	CCT	CAC	CCC	AGA	GGC	TAC	TAT	AAA	TGT	AGC	AGC	ATG	900
286	K	G	S	P	H	P	R	G	Y	Y	K	C	S	S	M	300
901	AGA	GGC	TGC	CCT	GCA	AGG	AAA	CAT	GTG	GAG	AGG	TGC	TTG	GAA	GAT	945
301	R	G	C	P	A	R	K	H	V	E	R	C	L	E	D	315
946	CCT	TCT	ATG	CTT	ATT	GTC	ACT	TAT	GAA	GGC	GAG	CAT	AAC	CAC	CCA	990
316	P	S	M	L	I	V	T	Y	E	G	E	H	N	H	P	330
991	AGG	ATA	CCA	GTC	CAA	TCA	ACA	ACC	ACA	TGA	1020					
331	R	I	P	V	Q	S	T	T	T	*						

Figure S1. *Cont.*

PgWRKY6

1	ATG	GAG	TAC	TAC	AAT	AGA	TTT	GTG	CAC	GAT	CAA	GAT	GAT	TCC	CCG	45
1	M	E	Y	Y	N	R	F	V	H	D	Q	D	D	S	P	15
46	GAA	ACT	GCC	TCT	GGC	TCT	CCA	CTT	TCC	GGC	GAG	GAT	ACC	ATT	ATG	90
16	E	T	A	S	G	S	P	L	S	G	E	D	T	I	M	30
91	GCC	GAT	ACC	CCG	TCA	CCC	AAG	AAA	AGT	AGG	AGG	ATT	GCA	GGG	AAG	135
31	A	D	T	P	S	P	K	K	S	R	R	I	A	G	K	45
136	AGA	GTG	GTG	ACA	GTG	GCA	ATA	GCC	GAT	GGG	GAT	GTA	TAT	CCA	CCT	180
46	R	V	V	T	V	A	I	A	D	G	D	V	Y	P	P	60
181	GCT	GAT	TCG	TGG	GCT	TGG	AGA	AAA	TAT	GGA	CAA	AAA	CCG	ATC	AAA	225
61	A	D	S	W	A	W	R	K	Y	G	Q	K	P	I	K	75
226	GGT	TCA	CCT	AAT	CCC	AGG	GGA	TAC	TAC	CGG	TGT	AGC	AGT	TCA	AAA	270
76	G	S	P	N	P	R	G	Y	Y	R	C	S	S	S	K	90
271	GGC	TGT	CCG	GCA	AGA	AAA	CAA	GTA	GAG	AGG	AGT	CGA	AAA	GAC	CCC	315
91	G	C	P	A	R	K	Q	V	E	R	S	R	K	D	P	105
316	ACC	GTG	GTT	GTA	ATC	ACC	TAT	GCT	TGT	GAA	CAC	AAC	CAC	CTC	ATT	360
106	T	V	V	V	I	T	Y	A	C	E	H	N	H	L	I	120
361	CCC	ACC	ACC	ACC	AAA	CAC	TCT	CAA	CCC	ACC	ATT	CCC	GTC	AAG	TTT	405
121	P	T	T	T	K	H	S	Q	P	T	I	P	V	K	F	135
406	CCA	CCA	GAA	GAA	GTC	GTG	GTT	TTT	GCC	AAC	CAG	ACA	GAC	CTT	GAA	450
136	P	P	E	E	V	V	V	F	A	N	Q	T	D	L	E	150
451	CCT	GAC	AAC	ATA	GAC	TTT	GCC	GAG	TTC	GTT	GCT	GAT	TTT	GGC	TAT	495
151	P	D	N	I	D	F	A	E	F	V	A	D	F	G	Y	165
496	TTC	ACC	AAC	ACA	ACG	TCT	GTC	ATA	CTA	GAG	AGC	ACT	GTA	ATT	ACA	540
166	F	T	N	T	T	S	V	I	L	E	S	T	V	I	T	180
541	AGC	CCC	AGA	TGC	ATG	GAA	CCC	GAT	TCA	GCA	GTG	ATT	TTC	ACA	AGG	585
181	S	P	R	C	M	E	P	D	S	A	V	I	F	T	R	195
586	GGA	GAT	GAT	GAG	GAT	TCC	TTG	TTT	GCT	GAC	CTT	GGT	GAG	CTA	CCG	630
196	G	D	D	E	D	S	L	F	A	D	L	G	E	L	P	210
631	GGA	TGT	TCA	CTA	ATT	TTT	CAG	CAG	TAA	657						
211	G	C	S	L	I	F	Q	Q	*							

Figure S1. *Cont.*

PgWRKY7

1	ATG	ACA	AAG	AGT	GAG	ATT	GAT	AAC	TTG	GAT	GAT	GGA	TAT	AGA	TGG	45
1	M	T	K	S	E	I	D	N	L	D	D	G	Y	R	W	15
46	AGG	AAG	TAC	GGC	CAA	AAA	GCT	GTC	AAA	AAC	AGC	CCT	TTC	CCA	AGG	90
16	R	K	Y	G	Q	K	A	V	K	N	S	P	F	P	R	30
91	AGC	TAC	TAT	CGT	TGC	ACT	ACT	GCA	GCC	TGT	GGT	GTG	AAA	AAG	AGG	135
31	S	Y	Y	R	C	T	T	A	A	C	G	V	K	K	R	45
136	GTT	GAA	AGA	TCA	TCC	GAT	GAT	CCC	TCC	ATT	GTT	GTT	ACA	ACC	TAT	180
46	V	E	R	S	S	D	D	P	S	I	V	V	T	T	Y	60
181	GAA	GGT	ACA	CAC	ACG	CAT	CCC	TGC	CCC	GTG	ACC	CCA	CGT	GGA	AGC	225
61	E	G	T	H	T	H	P	C	P	V	T	P	R	G	S	75
226	ATT	GGA	ATC	TTA	CCG	GAA	ACT	TCC	GCT	TTC	GGC	GGC	ATT	GCT	GGG	270
76	I	G	I	L	P	E	T	S	A	F	G	G	I	A	G	90
271	GGT	GGT	ACC	GGT	TCT	ATT	GGC	GGT	GAT	GCA	ATT	AAT	TCG	CCT	TAT	315
91	G	G	T	G	S	I	G	G	D	A	I	N	S	P	Y	105
316	GCC	GTT	CCC	CAG	CTT	CAC	TAT	CAA	CAA	ATA	CTA	ATG	CTG	CAG	CAG	360
106	A	V	P	Q	L	H	Y	Q	Q	I	L	M	L	Q	Q	120
361	CAA	CAA	CAA	CAA	AAC	CCC	TAT	TTA	TAT	AAT	AAC	TCG	ACA	TTA	TCT	405
121	Q	Q	Q	Q	N	P	Y	L	Y	N	N	S	T	L	S	135
406	TCC	TTC	AAC	TTT	AGA	GCT	ACT	AAT	ATC	AGT	ACT	ACT	AGT	ACT	AAT	450
136	S	F	N	F	R	A	T	N	I	S	T	T	S	T	N	150
451	CCG	GTC	TCG	TTT	CCT	AAT	CTT	TTT	CTT	CAA	GAC	CGA	CGT	TTT	TCC	495
151	P	V	S	F	P	N	L	F	L	Q	D	R	R	F	S	165
496	CCT	AAT	CCT	TCC	GCT	TTG	TTT	AGA	GAT	CAG	GGT	CTT	CTT	CAG	GAC	540
166	P	N	P	S	A	L	F	R	D	Q	G	L	L	Q	D	180
541	ATG	GTG	CCG	TCC	CAA	ATG	CGA	GTG	GAA	AAG	AAA	GAG	GAT	CAA	TGA	585
181	M	V	P	S	Q	M	R	V	E	K	K	E	D	Q	*	

Figure S1. *Cont.*

PgWRKY8

1	ATG	GAT	AAG	TAC	TCT	CCC	TCC	CCT	ATA	TTG	AAC	TCT	GCA	GAA	ACT	45
1	M	D	K	Y	S	P	S	P	I	L	N	S	A	E	T	15
46	CAG	GCT	TCC	AAA	AAA	CGG	AAG	ATG	TCT	CAG	AAG	ACT	GTA	TTA	ACA	90
16	Q	A	S	K	K	R	K	M	S	Q	K	T	V	L	T	30
91	GTG	AAG	ATT	GAA	GAA	AAT	GAA	AAT	GGG	AAA	CAG	AAG	AGT	GAG	GGG	135
31	V	K	I	E	E	N	E	N	G	K	Q	K	S	E	G	45
136	CCT	CCT	CCT	TCT	GAT	TGT	TGG	TCA	TGG	AGG	AAA	TAT	GGG	CAG	AAA	180
46	P	P	P	S	D	C	W	S	W	R	K	Y	G	Q	K	60
181	CCC	ATC	AAA	GGA	TCT	CCT	TAT	CCC	AGG	GGA	TAC	TAC	AAA	TGC	AGC	225
61	P	I	K	G	S	P	Y	P	R	G	Y	Y	K	C	S	75
226	ACA	TCA	AAG	GGT	TGT	TCA	GCC	AAA	AAA	CAA	GTA	GAA	AGA	TGC	AGA	270
76	T	S	K	G	C	S	A	K	K	Q	V	E	R	C	R	90
271	ACA	GAT	GCT	TCA	TTG	CTC	ATC	GTC	ACT	TAC	ACC	TCT	ACT	CAT	AAC	315
91	T	D	A	S	L	L	I	V	T	Y	T	S	T	H	N	105
316	CAT	CCA	AGT	CCC	AAA	GAG	CCA	AAA	CAA	GAA	CAA	CCC	AAC	ATC	CAA	360
106	H	P	S	P	K	E	P	K	Q	E	Q	P	N	I	Q	120
361	ATC	ACC	GAG	GAA	GAC	AAC	AGT	CTG	ATA	ACC	CCA	GAA	GAA	AAA	GAT	405
121	I	T	E	E	D	N	S	L	I	T	P	E	E	K	D	135
406	GAA	CAA	TCC	CTC	AAT	GGT	CAT	GAA	GAA	GAT	GGA	GCT	AGT	GAA	AAT	450
136	E	Q	S	L	N	G	H	E	E	D	G	A	S	E	N	150
451	ATT	ACT	GAT	TTT	CAC	TAC	TGC	CAA	TCT	CCA	TTC	AAT	AGC	TCT	GAT	495
151	I	T	D	F	H	Y	C	Q	S	P	F	N	S	S	D	165
496	CAT	CAG	GAC	ATC	GTC	ATT	AAC	ATC	AGA	CAT	GAA	GAA	GAA	AGC	ACT	540
166	H	Q	D	I	V	I	N	I	R	H	E	E	E	S	T	180
541	TTT	ACA	GAA	AAT	CTA	CAG	ACT	GTC	CTG	TTT	GAT	GAA	AAA	GAG	AAA	585
181	F	T	E	N	L	Q	T	V	L	F	D	E	K	E	K	195
586	CCC	CTT	TGT	TAT	CCT	CAT	CTC	ATG	ACC	TTC	TCA	ACC	CCC	AAA	TCA	630
196	P	L	C	Y	P	H	L	M	T	F	S	T	P	K	S	210
631	GAA	GAA	AAT	GAC	TTC	TAT	GAT	GAG	CTT	GGA	GAA	CTA	CCC	CCA	TCT	675
211	E	E	N	D	F	Y	D	E	L	G	E	L	P	P	S	225
676	ACT	TCT	TAT	TTT	ACA	AGC	TTC	ATG	AGG	GGA	AAT	TTC	TTT	GAG	GAT	720
226	T	S	Y	F	T	S	F	M	R	G	N	F	F	E	D	240
721	AGG	ATT	CTT	GTT	CAC	CTA	TCT	TGA	744							
241	R	I	L	V	H	L	S	*								

Figure S1. *Cont.*

PgWRKY9

1	ATG	GAC	GGC	AGA	ATC	AAT	CGG	TTT	ATG	AAT	GAG	CAA	GAA	GAG	TAT	45
1	M	D	G	R	I	N	R	F	M	N	E	Q	E	E	Y	15
46	GAT	AAT	TCA	CCG	GAA	AAC	AGC	GGA	GAT	TCA	CCG	CCT	TCT	TCC	ATG	90
16	D	N	S	P	E	N	S	G	D	S	P	P	S	S	M	30
91	TTT	AGT	GAC	ACC	AAG	ATG	GCT	TCC	ACT	TCC	TCT	CCG	AGA	AGA	AGT	135
31	F	S	D	T	K	M	A	S	T	S	S	P	R	R	S	45
136	AGG	AGA	GGT	ATG	CAG	AAA	AGA	GTG	GTG	TCC	GTG	CCA	ATC	AAA	GAC	180
46	R	R	G	M	Q	K	R	V	V	S	V	P	I	K	D	60
181	GTC	GAA	AGG	TCC	AAG	CTT	AAA	GGG	GAG	ATG	GCG	AGT	ACT	CCA	CCG	225
61	V	E	R	S	K	L	K	G	E	M	A	S	T	P	P	75
226	TCA	GAT	TCT	TGG	GCT	TGG	AGA	AAG	TAT	GGT	CAA	AAG	CCT	ATT	AAA	270
76	S	D	S	W	A	W	R	K	Y	G	Q	K	P	I	K	90
271	GGA	TCT	CCT	TAC	CCC	AGG	GGT	TAT	TAT	AGA	TGT	AGT	AGC	TCA	AAA	315
91	G	S	P	Y	P	R	G	Y	Y	R	C	S	S	S	K	105
316	GGT	TGC	CCG	GCA	AGA	AAA	CAA	GTC	GAG	AGG	AGC	CGG	GCG	GAT	CCC	360
106	G	C	P	A	R	K	Q	V	E	R	S	R	A	D	P	120
361	ACC	ACA	CTA	TTG	GTG	ACC	TAC	TCT	TGT	GAC	CAC	AAT	CAT	CCC	GGG	405
121	T	T	L	L	V	T	Y	S	C	D	H	N	H	P	G	135
406	CCG	GCT	TCT	AGA	AAC	AAC	AAC	CGC	AAG	CAC	AAT	CGC	AGT	CGC	AAT	450
136	P	A	S	R	N	N	N	R	K	H	N	R	S	R	N	150
451	AAT	AAT	CAA	GAT	ATT	ACT	ACT	ACC	TCC	GCC	TCC	GCC	TCC	GCG	AGT	495
151	N	N	Q	D	I	T	T	T	S	A	S	A	S	A	S	165
496	AAT	ATT	ACT	ACT	TCA	TCA	ACA	AAC	CTC	ACA	ATT	TCA	AAT	TCT	GAA	540
166	N	I	T	T	S	S	T	N	L	T	I	S	N	S	E	180
541	GAT	GAT	CAA	ATA	TCA	GAA	TTT	TTT	GAG	ACC	GAT	GAA	AAA	TTC	AAT	585
181	D	D	Q	I	S	E	F	F	E	T	D	E	K	F	N	195
586	AAT	CTC	GAA	GGC	TCC	TTG	ATT	TCG	GAA	TTC	GGG	TGG	TTT	TCC	GGT	630
196	N	L	E	G	S	L	I	S	E	F	G	W	F	S	G	210
631	TTG	GAG	TCC	ACT	TCT	TCC	ACC	ATG	CTA	GAA	AGC	CCA	TTC	TTG	GCT	675
211	L	E	S	T	S	S	T	M	L	E	S	P	F	L	A	225
676	AGA	GAC	TGC	ATT	GGA	GAT	GCT	GAC	ATG	GCA	ACA	ATT	TTT	TCA	ATG	720
226	R	D	C	I	G	D	A	D	M	A	T	I	F	S	M	240
721	GGG	GAG	GAG	GAA	GAG	TCA	TTG	TTC	GCC	GAT	CTC	GGG	GAG	CTG	CCG	765
241	G	E	E	E	E	S	L	F	A	D	L	G	E	L	P	255
766	GAG	TGT	TCG	GTG	GTA	TTC	CGG	CCA	GGG	GTG	ATG	GAG	AGA	GAG	GAG	810
256	E	C	S	V	V	F	R	P	G	V	M	E	R	E	E	270
811	GAG	CGC	CGG	CGG	TGT	AGC	TTG	ACA	ACC	TTG	TAG	843				
271	E	R	R	R	C	S	L	T	T	L	*					

Figure S1. *Cont.*

PgWRKY2

1	ATG	GAG	GTT	GAA	GAA	GCT	AAC	AAA	GCA	GCA	GTT	GAG	AGT	TGC	CAC	45
1	M	E	V	E	E	A	N	K	A	A	V	E	S	C	H	15
46	AGA	GTT	CTA	AGT	CTG	ATA	TCC	CAG	CCC	CAA	GAT	CAG	ATT	CAG	TAT	90
16	R	V	L	S	L	I	S	Q	P	Q	D	Q	I	Q	Y	30
91	AGG	AAT	TTA	GTG	GTT	GAA	ACT	GGG	GAA	GCT	GTA	CTT	AAG	TTT	GAA	135
31	R	N	L	V	V	E	T	G	E	A	V	L	K	F	E	45
136	AAA	GTT	GTT	TCT	CTT	CTT	AAT	GCT	GGT	TTG	GGG	CAT	GCA	AGA	GTG	180
46	K	V	V	S	L	L	N	A	G	L	G	H	A	R	V	60
181	AGA	AAG	ATC	GAG	AAA	ATT	CAA	ACC	CCT	TTT	CCC	CAA	AAC	ATA	CTC	225
61	R	K	I	E	K	I	Q	T	P	F	P	Q	N	I	L	75
226	TTA	GAC	AAC	CCA	ATT	GGT	AGA	CCT	GAT	TAT	CAA	CCG	AAA	GCG	ATT	270
76	L	D	N	P	I	G	R	P	D	Y	Q	P	K	A	I	90
271	CAG	CTT	CTT	TCA	GCT	AAT	TCC	CTT	GAC	ACC	CCA	ATT	CAT	GAT	AAG	315
91	Q	L	L	S	A	N	S	L	D	T	P	I	H	D	K	105
316	GGT	TCA	AAT	GTT	CGA	AGT	ACT	CTT	ACT	TTA	GGA	AAT	TCA	TCA	TTG	360
106	G	S	N	V	R	S	T	L	T	L	G	N	S	S	L	120
361	GAA	CTG	AGT	TCA	AAT	GGG	AAA	AAC	TCT	CTT	CAA	ATA	CCC	CAA	CAA	405
121	E	L	S	S	N	G	K	N	S	L	Q	I	P	Q	Q	135
406	ACA	CCA	CCA	TCA	AAC	TAT	CAC	TTC	CTG	CAG	CAA	AAG	TTT	CAG	CTT	450
136	T	P	P	S	N	Y	H	F	L	Q	Q	K	F	Q	L	150
451	CAG	CAG	CAA	CAG	TTA	AAA	CAG	CAA	GCT	GAA	ATG	ATG	TTC	CGT	CGG	495
151	Q	Q	Q	Q	L	K	Q	Q	A	E	M	M	F	R	R	165
496	AGC	AAT	AGT	GGC	ATC	AAC	CTG	AAT	TTT	GAT	AGC	TCT	ACC	TGC	ACG	540
166	S	N	S	G	I	N	L	N	F	D	S	S	T	C	T	180
541	CCC	ACC	ATG	TCA	TCC	ACT	AGG	TCA	TTT	ATC	TCC	TCG	TTG	AGT	GTC	585
181	P	T	M	S	S	T	R	S	F	I	S	S	L	S	V	195
586	GAT	GGG	AGT	GTG	GCT	AAT	ATG	GAG	GGC	AAT	GCT	TTC	CAT	TTA	ATT	630
196	D	G	S	V	A	N	M	E	G	N	A	F	H	L	I	210
631	GGT	GCG	ACT	CGC	TCT	TTG	GAT	CAG	AGT	TCA	TAC	CAG	CAC	AAA	CGA	675
211	G	A	T	R	S	L	D	Q	S	S	Y	Q	H	K	R	225
676	AGG	TGC	TCT	GCA	AAG	GGA	GAT	GAT	GGC	AGT	GTG	AAG	TGT	GGA	AGC	720
226	R	C	S	A	K	G	D	D	G	S	V	K	C	G	S	240
721	AGT	GGT	AGA	TGT	CAC	TGC	TCA	AAG	AAG	AGG	AAA	CAT	AGG	GTA	AAG	765
241	S	G	R	C	H	C	S	K	K	R	K	H	R	V	K	255
766	AGA	TCA	ATC	AAA	GTA	CCT	GCT	ATA	AGC	AGC	AAG	CTC	GCT	GAC	ATC	810
256	R	S	I	K	V	P	A	I	S	S	K	L	A	D	I	270
811	CCT	CCT	GAT	GAG	TAT	TCA	TGG	AGG	AAG	TAT	GGT	CAG	AAA	CCA	ATC	855
271	P	P	D	E	Y	S	W	R	K	Y	G	Q	K	P	I	285
856	AAG	GGT	TCT	CCT	CAC	CCC	AGA	GGC	TAC	TAT	AAA	TGC	AGC	AGC	ATG	900
286	K	G	S	P	H	P	R	G	Y	Y	K	C	S	S	M	300
901	AGA	GGC	TGC	CCT	GCA	AGG	AAA	CAT	GTG	GAG	AGG	TGC	TTG	GAA	GAT	945
301	R	G	C	P	A	R	K	H	V	E	R	C	L	E	D	315
946	CCT	TCT	ATG	CTT	ATT	GTC	ACT	TAT	GAA	GGC	GAG	CAT	AAC	CAC	CCA	990
316	P	S	M	L	I	V	T	Y	E	G	E	H	N	H	P	330
991	AGG	ATA	CCA	GTC	CAA	TCA	ACA	ACC	ACA	TGA	1020					
331	R	I	P	V	Q	S	T	T	T	*						

Figure S1. *Cont.*

PgWRKY3

1	ATG	ACT	GTT	GAC	CTG	ATG	AGC	AGT	GGT	TAC	AAT	TTT	GGT	GCC	AAA	45
1	M	T	V	D	L	M	S	S	G	Y	N	F	G	A	K	15
46	ATG	GAA	GAA	ACC	ACC	GTC	CAA	GAA	GCT	GCC	ACC	GCC	GGA	CTT	CAG	90
16	M	E	E	T	T	V	Q	E	A	A	T	A	G	L	Q	30
91	AGC	GTC	GAG	AAT	CTA	ATC	AGG	ATT	CTT	TCT	CAC	TCC	TCC	CAA	CAA	135
31	S	V	E	N	L	I	R	I	L	S	H	S	S	Q	Q	45
136	TTT	CAC	AAC	CAA	AAC	CCA	ACA	AAT	CAC	TCA	TCT	TTT	TCC	TCA	ACC	180
46	F	H	N	Q	N	P	T	N	H	S	S	F	S	S	T	60
181	TCC	ATG	GAT	TCC	GGC	AAT	ACC	GAT	TAC	AGA	GCT	GTA	ACA	GAT	ATG	225
61	S	M	D	S	G	N	T	D	Y	R	A	V	T	D	M	75
226	GCA	GTG	AAC	AAA	TTC	AAA	AAG	TTC	ATT	TCT	CTG	CTC	GAC	CGG	ACC	270
76	A	V	N	K	F	K	K	F	I	S	L	L	D	R	T	90
271	CGA	ACC	GGT	CAC	GCA	CGG	TTC	AGA	AGA	GGC	CCA	ATC	GTC	CAC	CAC	315
91	R	T	G	H	A	R	F	R	R	G	P	I	V	H	H	105
316	CAA	CAG	CAT	CAA	CAG	CGG	CCG	GAA	ACC	CAG	ACT	CAC	GAA	TCA	GAG	360
106	Q	Q	H	Q	Q	R	P	E	T	Q	T	H	E	S	E	120
361	CCG	TTG	ATT	CAG	TTA	AAT	GGG	CAT	CAA	AAC	CAC	CAT	CAC	CAC	CAT	405
121	P	L	I	Q	L	N	G	H	Q	N	H	H	H	H	H	135
406	CAG	ACG	GTG	GAG	AAG	GAA	ATG	ATG	TCT	AAT	GGG	TCC	CGA	ATT	TAT	450
136	Q	T	V	E	K	E	M	M	S	N	G	S	R	I	Y	150
451	TGT	CCC	ACT	CCG	GTT	CAG	CGC	TTA	CCT	CCG	CCG	GTT	CTC	AAT	AAC	495
151	C	P	T	P	V	Q	R	L	P	P	P	V	L	N	N	165
496	AAG	CAT	CAT	CAT	CAG	TTA	GTA	AAA	AAT	GGA	TCT	ATT	GAA	AAG	AAG	540
166	K	H	H	H	Q	L	V	K	N	G	S	I	E	K	K	180
541	GAA	CCA	ATA	ACC	ACC	ATT	AAC	TTT	GCT	CCG	GTC	GCG	TTG	ACG	ACG	585
181	E	P	I	T	T	I	N	F	A	P	V	A	L	T	T	195
586	GTG	TCG	CCG	GCG	ACT	TCG	TTT	ATG	TCA	TCG	TTG	ACC	GGA	GAT	ACG	630
196	V	S	P	A	T	S	F	M	S	S	L	T	G	D	T	210
631	GAT	GGG	TCG	GGT	TTT	CAG	ATC	ACG	AAT	ATA	TCG	CAG	GTC	TCG	TCT	675
211	D	G	S	G	F	Q	I	T	N	I	S	Q	V	S	S	225
676	GGT	AGC	CGG	CCG	CCG	CTT	TCT	TCG	TCG	TCG	TTT	AAG	CGG	AAG	TGC	720
226	G	S	R	P	P	L	S	S	S	S	F	K	R	K	C	240
721	AGT	TCG	ATG	GAT	GAT	TCT	GCG	GCA	AAG	TGC	TCT	GGG	TCT	TCC	GGC	765
241	S	S	M	D	D	S	A	A	K	C	S	G	S	S	G	255
766	AGA	TGC	TAT	TGT	CCT	AAG	AAA	AGG	AAA	TCA	AGG	ATG	AAG	AAT	GTG	810
256	R	C	Y	C	P	K	K	R	K	S	R	M	K	N	V	270
811	GTG	AGA	GTT	CCG	GCC	ATA	AGT	ATG	AAG	ATG	TCT	GAT	ATC	CCA	CCG	855
271	V	R	V	P	A	I	S	M	K	M	S	D	I	P	P	285
856	GAT	GAT	TTT	TCT	TGG	AGA	AAG	TAT	GGT	CAA	AAG	CCC	ATC	AAA	GGT	900
286	D	D	F	S	W	R	K	Y	G	Q	K	P	I	K	G	300
901	TCT	CCC	CAT	CCA	AGG	GGA	TAT	TAC	AAG	TGC	AGT	AGC	GTA	AGA	GGG	945
301	S	P	H	P	R	G	Y	Y	K	C	S	S	V	R	G	315
946	TGC	CCG	GCT	AGA	AAG	CAT	GTA	GAG	AGG	GCA	GTG	GAT	GAT	CCG	GCA	990
316	C	P	A	R	K	H	V	E	R	A	V	D	D	P	A	330
991	ATG	TTG	GTG	GTG	ACC	TAC	GAG	GGG	GAG	CAC	AAC	CAT	TCT	CGC	TCT	1035
331	M	L	V	V	T	Y	E	G	E	H	N	H	S	R	S	345
1036	ATT	AAC	GAC	ACA	CCG	GCA	TCG	CTA	GTT	CTT	GAA	TCG	TCT	TAA		1077
346	I	N	D	T	P	A	S	L	V	L	E	S	S	*		

Figure S1. *Cont.*

PgWRKY4

1	ATG	ACT	GTT	GAC	CTG	ATG	AGC	AGC	GGT	TAC	AAT	TTT	GGT	GCC	AAA	45
1	M	T	V	D	L	M	S	S	G	Y	N	F	G	A	K	15
46	ATG	GAA	GAA	ACC	ACC	GTT	CAA	GAA	GCT	GCC	ACC	GCC	GGA	CTT	CAG	90
16	M	E	E	T	T	V	Q	E	A	A	T	A	G	L	Q	30
91	AGC	GTC	GAG	AAT	CTC	ATC	AGG	GTT	CTT	TCT	CAC	TCC	TCC	CAA	CAA	135
31	S	V	E	N	L	I	R	V	L	S	H	S	S	Q	Q	45
136	TTT	CAC	AAC	CAA	AAC	CCA	ACA	AAT	CAC	TCA	TCT	TCT	TCC	TCA	ACC	180
46	F	H	N	Q	N	P	T	N	H	S	S	S	S	S	T	60
181	TCC	ATG	GAT	TCC	GGC	AAT	ACC	GAT	TAC	AGA	GCT	GTA	ACA	GAT	ATG	225
61	S	M	D	S	G	N	T	D	Y	R	A	V	T	D	M	75
226	GCA	GTG	AAC	AAA	TTC	AAA	AAG	TTC	ATT	TCT	CTG	CTC	GAC	CGG	ACC	270
76	A	V	N	K	F	K	K	F	I	S	L	L	D	R	T	90
271	CGA	ACC	GGT	CAC	GCA	CGA	TTC	AGA	AGA	GGC	CCA	ATC	GTC	CAC	CAC	315
91	R	T	G	H	A	R	F	R	R	G	P	I	V	H	H	105
316	CAA	CAG	CAT	CAA	CAG	CGG	CCG	GAA	ACC	CAG	ACT	CAC	GAA	TCA	GAG	360
106	Q	Q	H	Q	Q	R	P	E	T	Q	T	H	E	S	E	120
361	CCG	TTG	ATT	CAG	TTA	AAT	GGG	CAT	CAA	AAC	CAC	CAC	CAC	CAC	CAT	405
121	P	L	I	Q	L	N	G	H	Q	N	H	H	H	H	H	135
406	CAG	ACG	GTG	GAG	AAG	GAA	ATG	ATG	TCT	AAT	GGG	TCC	CGA	ATT	TAT	450
136	Q	T	V	E	K	E	M	M	S	N	G	S	R	I	Y	150
451	TGT	CCC	ACT	CCG	GTT	CAG	CGC	TTA	CCT	CCG	CCG	GTT	CTC	AAT	AAC	495
151	C	P	T	P	V	Q	R	L	P	P	P	V	L	N	N	165
496	AAG	CAT	CAT	CAT	CAG	TTA	GTG	AAA	AAT	GGA	TCT	ATT	GAA	AAG	AAG	540
166	K	H	H	H	Q	L	V	K	N	G	S	I	E	K	K	180
541	GAA	CCA	ATA	ACC	ACC	ATT	AAT	TTT	GCA	CCG	GTC	GCG	TTG	ACG	GCG	585
181	E	P	I	T	T	I	N	F	A	P	V	A	L	T	A	195
586	GTG	TTG	CCG	GCG	ACC	TCG	TTT	ATG	TCG	TCG	TTG	ACC	GGA	GAT	ACG	630
196	V	L	P	A	T	S	F	M	S	S	L	T	G	D	T	210
631	GAT	GGG	TCG	GGT	TTT	CAG	ATC	ACG	AAT	ATA	TCG	CAG	GTC	TCG	TCG	675
211	D	G	S	G	F	Q	I	T	N	I	S	Q	V	S	S	225
676	GGT	AGC	CGG	CCG	CCG	CTT	TCT	TCT	TCG	TCG	TTC	AAG	CGG	AAG	TGC	720
226	G	S	R	P	P	L	S	S	S	S	F	K	R	K	C	240
721	AGT	TCG	ATG	GAT	GAT	TCT	GCG	GCT	AAG	TGC	TCT	GGG	TCT	TCC	GGC	765
241	S	S	M	D	D	S	A	A	K	C	S	G	S	S	G	255
766	AGA	TGC	CAT	TGT	CCT	AAG	AAA	AGG	AAA	TCA	AGG	ATG	AAG	AAT	GTG	810
256	R	C	H	C	P	K	K	R	K	S	R	M	K	N	V	270
811	GTG	AGA	GTT	CCG	GCC	ATA	AGT	ATG	AAG	ATG	TCT	GAT	ATC	CCA	CCG	855
271	V	R	V	P	A	I	S	M	K	M	S	D	I	P	P	285
856	GAT	GAT	TTT	TCT	TGG	AGA	AAG	TAT	GGT	CAA	AAG	CCC	ATC	AAA	GGT	900
286	D	D	F	S	W	R	K	Y	G	Q	K	P	I	K	G	300
901	TCT	CCC	CAT	CCA	AGA	GGA	TAT	TAC	AAG	TGC	AGT	AGC	GTA	AGA	GGG	945
301	S	P	H	P	R	G	Y	Y	K	C	S	S	V	R	G	315
946	TGC	CCG	GCT	AGA	AAG	CAT	GTA	GAG	AGG	GCA	GTG	GAT	GAT	CCG	GCA	990
316	C	P	A	R	K	H	V	E	R	A	V	D	D	P	A	330
991	ATG	TTG	GTA	GTG	ACC	TAC	GAG	GGG	GAG	CAC	AAC	CAT	TCT	CGC	GCT	1035
331	M	L	V	V	T	Y	E	G	E	H	N	H	S	R	A	345
1036	ATT	AAC	GAC	ACA	CCG	GCA	TCG	CTA	GTT	CTT	GAA	TCG	TCT	TAA		1077
346	I	N	D	T	P	A	S	L	V	L	E	S	S	*		

Figure S1. Nucleotide and amino acid sequences of the eight studied *PgWRKYs* (*PgWRKY2*, *PgWRKY3*, *PgWRKY4*, *PgWRKY5*, *PgWRKY6*, *PgWRKY7*, *PgWRKY8*, and *PgWRKY9*). The nucleotides are presented in the upper line and the amino acid residues are shown below. The WRKY motifs are underlined with pink color and cysteine (C) and histidine (H) residues in the putative zinc-finger motifs with blue color.

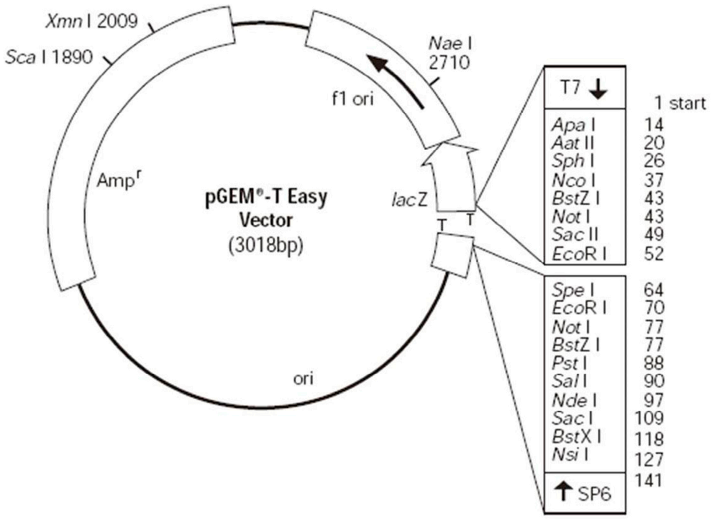

Figure S2. The pGEM-T Easy vector used for cloning. The arrow located inside the loop indicates the single strand phage DNA (for DNA sequencing) replication and the arrow present outside indicates multiple cloning sites.
